# Physical illnesses among psychiatric outpatients in a tertiary care health institution: A prospective study

**DOI:** 10.4103/0019-5545.31620

**Published:** 2006

**Authors:** Gurvinder Pal Singh, B.S. Chavan, Paramleen Kaur, Shalini Bhatia

**Affiliations:** * Senior Lecturer, Department of Psychiatry, Government Medical College and Hospital, Sector 32, Chandigarh; ** Professor and Head, Department of Psychiatry, Government Medical College and Hospital, Sector 32, Chandigarh; *** Senior Resident, Department of Psychiatry, Government Medical College and Hospital, Sector 32, Chandigarh; **** Medical Social Worker, Department of Psychiatry, Government Medical College and Hospital, Sector 32, Chandigarh

**Keywords:** General physical examination, physical illness, psychiatric patient

## Abstract

**Background:**

The recognition of physical illnesses by mental health professionals has important clinical implications.

**Aim:**

This study aimed to find the nature and prevalence of associated physical illnesses in psychiatric outpatients.

**Methods:**

Two hundred fifty consecutive psychiatric outpatients who fulfilled the inclusion criteria of the study were assessed in detail for associated physical illnesses. A conclusive physical diagnosis was based on the clinical history, general physical examination and investigation reports.

**Results:**

Forty-eight per cent of the patients were found to have associated physical illnesses. In about one-fifth of the total sample (*n*=51; 20.4%) the associated physical illness was diagnosed for the first time. Hypertension (29.1%), respiratory diseases (15%), anaemia (12.5%), diabetes mellitus (10%) and liver diseases (5.8%) were some common physical illnesses found in these patients.

**Conclusion:**

Common physical illnesses of psychiatric patients can be detected mostly by a careful history-taking checklist and physical examination. Psychiatrists must inculcate the habit of conducting a general physical examination of their psychiatric patients.

## INTRODUCTION

The prevalence of physical illness in psychiatric patients is higher than that in the general population. Both in developed and developing countries, very little attention is paid to the physical illness of psychiatric patients, which leads to a poorer outcome. In one survey, <35% of practising psychiatrists routinely examined their patients physically and 32% indicated that they did not feel competent to perform a physical examination. It was further reported that a physical examination is performed on only 13% of inpatients and 8% of psychiatric outpatients.^1^,^2^ Lack of routine physical examination by psychiatrists leads to decay of their clinical skills over a period of time. In another study, more than half the psychiatrists did not feel competent and some admitted that they did not like performing a physical examination.^3^

Studies reveal that simple referral of psychiatric patients to other physicians for examination of possible physical illnesses often proves insufficient.^4^ Reliance on the notion of ‘medical clearance’ of psychiatric patients by other physicians is not adequate unless the extent of the referring physician's evaluation is known.^5^ This is of increasing concern for mental health professionals in clinical practice. All major psychiatric disorders such as depressive disorders, schizophrenia and anxiety disorders have a close association with physical illnesses. In India, there is a paucity of literature concerning the prevalence of physical illnesses in psychiatric patients. As not much work has been done in this regard in the northern part of India, this study was carried out to examine the nature and prevalence of physical illnesses in patients attending psychiatry OPD services.

## MATERIAL AND METHODS

### Setting

This study was conducted in the walk-in clinic of the Department of Psychiatry, Government Medical College and Hospital, Chandigarh, which is a tertiary care health institution. On first contact, patients are seen in the walk-in clinic by a psychiatrist who makes a psychiatric diagnosis and initiates treatment.

### Sample

The study sample consisted of 400 consecutive subjects from the walk-in clinic. The study sample also included those patients who were referred to the psychiatry OPD from general medicine and other OPD services. Informed consent was sought from the subjects for conducting a physical examination and investigations.

The inclusion criteria for this study were as follows:

- Subjects should have a psychiatric diagnosis as per ICD-10 criteria (WHO, 1992)^6^
- Subjects should be willing to undergo investigations and other procedures needed for confirmation of a physical diagnosis.

A total of 381 subjects consented to participate in the study; 13 subjects who did not receive a psychiatric diagnosis and 118 subjects who did not come for follow-up visits had to be excluded. Hence, data were collected and analysed for 250 subjects.

### Tools

The following tools were used in this study:

1. *Semi-structured sociodemographic proforma:* This proforma was used to record a detailed sociodemographic profile of the subjects including age, sex, marital status, occupation, education, family type, locality and source of referral.
2. *Semi-structured proforma for making a physical diagnosis:* This proforma had three subcomponents:
  - History-taking questionnaire—consisting of 7 items related to common physical problems with yes/no responses
  - General physical examination proforma—used for recording positive clinical findings
  - Investigations battery—this included routine (haemo-gram and complete urine examination) and special investigations (renal, liver, thyroid function tests, ECG, EEG, X-ray and ultrasonography).
3. *Semi-structured proforma for psychiatric diagnosis:* This proforma was used for recording a concise clinical history and findings of the mental status examination.

### Procedure

All the subjects were initially assessed by a medical social worker (MSW) to fill in proforma no. 1. They were then interviewed and examined by the senior resident (psychiatrist) after obtaining informed consent, and proforma nos. 2 and 3 were completed. All the subjects underwent routine investigations. Based on the history and physical examination, the need for further investigations was assessed in consultation with specialists. As per the hospital policy, on being recommended by both the Head of the Department and Medical Superintendent, poor people could undergo investigations free of charge. Psychiatric and physical diagnoses were made by the psychiatrist and subjects were referred to the concerned specialists. The subjects were divided into three groups:

**Table:** 

Group 1:	Subjects with freshly diagnosed physical illness
Group 2:	Subjects with previously diagnosed physical illness, before being referred to the psychiatry OPD
Group 3:	Subjects without any physical illness

Before enrolling the subjects, the psychiatrists rehearsed their clinical examination skills under the supervision of specialists from various departments. The data were analysed using the Jandel SigmaStat version 2 statistical software.^7^ Descriptive statistical analysis, analysis of variance, chi-square and McNemar tests^8^ were used.

## RESULTS

The sociodemographic characteristics of the subjects are summarized in Table 1. The majority of the subjects were male (63.2%), married (68.8%) and belonged to urban areas. The age range of the subjects was 9–75 years (34.54±13.24 years). Subjects with previously undiagnosed physical illnesses (group 2) were significantly older, more often married, unemployed, less educated and belonged to the lower income group. One-third of these subjects had been referred from the Medical Outdoor Patient Department (MOPD), i.e. the physical diagnosis was missed in the MOPD. About one-fifth of the total sample (group 1, *n*=51; 20.4%) were detected for the first time to have an associated physical illness (Table 1). Results of routine investigations were within normal limits for 175 patients.

**Table 1:** Sociodemographic profile of the subjects (*n*=250)

Variable	Variants	Group 1 *n* (%)		Group 2 *n* (%)		Group 3 *n* (%)		Significance	
			
		51	(20.4)	69	(27.6)	130	(52)		
Age (in years)	Mean±SD	41.5±12.8		38.9±14.2		30.3±10.9		χ2=36.270	S
	Range	14-75		9-70		14-62		df=2	p<0.001*
Sex	Male	35	(68.6)	41	(59.4)	82	(63.1)	χ2=1.071	NS
	Female	16	(31.4)	28	(40.5)	48	(36.9)	df=2	p=0.585
Marital status	Single	10	(19.6)	13	(18.8)	50	(38.5)	χ2=10.466	S
	Married	40	(78.4)	55	(79.7)	77	(59.2)	df=4	p=0.033*
	Others^1^	1	(1.9)	1	(1.4)	3	(2.3)		
Occupation	Employed	27	(52.9)	27	(39.1)	51	(39.1)	χ2=10.962	S
	Unemployed	11	(21.6)	7	(10.1)	15	(11.5)	df=2	p=0.004*
	Others^2^	13	(25.5)	35	(50.7)	63	(48.4)		
Education	Up to matric	40	(78.4)	40	(58)	67	(51.6)	χ2=11.057	S
	Above matric	11	(21.6)	29	(42)	63	(48.4)	df=4	p=0.026*
Income (Rupees per month)	0-3500	36	(70.6)	36	(52.2)	80	(61.5)	χ2=7.658	S
	3501-7000	13	(25.5)	18	(26.1)	22	(16.9)	df=2	p=0.022*
	>7000	2	(3.9)	15	(21.7)	28	(21.6)		
Locality	Urban	29	(56.9)	50	(72.5)	68	(52.3)	χ2=9.839	S
	Rural	22	(43.1)	19	(27.5)	62	(47.7)	df=4	p=0.043*
Referred from	Direct	22	(43.1)	31	(44.9)	81	(62.3)	χ2=134.884	S
	Medicine OPD	17	(33.3)	26	(37.7)	35	(26.9)	df=3	p<0.001*
	Others^3^	12	(23.5)	12	(17.4)	14	(10.8)		

**Note:** Group 1: Subjects with freshly diagnosed physical illness; Group 2: Subjects with previously diagnosed physical illness; Group 3: Subjects without physical illness

1 included widowed, remarried, divorced, separated persons;

2 included housewives, retired persons, students;

3 included secondary and primary care health centres, OPDs other than MOPD, non-governmental organizations, welfare agencies NS: non-sgnificant; S: significant; df: degree of freedom

Table 2 shows the diagnostic break-up of psychiatric disorders. The major diagnostic group was mood disorders (44.4%). Other groups were neurotic, stress-related and somatoform disorders (21.2%); schizophrenia, schizotypal and delusional disorders (13.2%); and substance use disorders (12%).

**Table 2:** Psychiatric diagnoses of the study subjects (*n*=250)

Diagnosis	*n* (%)
Mood (affective) disorders	111 (44.4)
Neurotic, stress-related and somatoform disorders	53 (21.2)
Schizophrenia, schizotypal and delusional disorders	33 (13.2)
Mental and behavioural disorders due to psychoactive substance use	30 (12)
Behavioural syndromes associated with physiological disturbances and physical factors	10 (4)
Organic, including symptomatic, mental disorders	7 (2.8)
Others	6 (2.4)

Table 3 shows the associated physical illnesses. Of the 250 subjects, 120 (48%) were found to have associated physical illnesses. Common physical illnesses were hypertension (29.1%), respiratory diseases (15%), anaemia (12.5%), diabetes mellitus (10%), liver diseases (5.8%), prolapsed intervertebral disc and spinal diseases (5%). Among hypertensive subjects taking beta-blockers, 12 out of 20 (60%) were diagnosed with depression whereas among the previously undiagnosed, treatment-naïve hypertensives, 10 out of 24 (42.6%) had depression. This difference is statistically non-significant (χ^2^=0.190, df=1, p=0.663). Of the 11 patients suffering from seizure disorder 1 (9.9%) was diagnosed with acute psychosis secondary to seizure disorder. In this study, 1 subject had depression secondary to hypothyroidism.

**Table 3:** Physical diagnoses of the study subjects (*n*=120)

Diagnosis	*n* (%)
Hypertension	35 (29.1)
Bronchial asthma	18 (15)
Anaemia	15 (12.5)
Diabetes mellitus	12 (10)
Epilepsy	11 (9.1)
Liver diseases	7 (5.8)
Eye diseases	6 (5)
Skin diseases	6 (5)
Prolapsed intervertebral disc and spinal diseases	6 (5)
Hypothyroidism	4 (3.3)

## DISCUSSION

The findings of this prospective hospital-based study have implications for clinical practice. In this study, a total of 48% (*n*=120) of the psychiatric outpatients were found to have physical illnesses. It is also relevant that the number of patients with illnesses increased from 69 (27.6%) to 120 (48%) with the systematic application of a history-taking checklist and physical examination. Other authors have also reported similar^1^,^9^ or even higher^10^–^12^ rates of prevalence of physical illnesses in psychiatric patients in a variety of settings.

Another significant finding of this study was that a large number of subjects (20.4%) had previously undiagnosed physical illnesses. In another study, where 41.3% of psychiatric patients had a medical problem, 65% of the diagnoses were previously unsuspected.^13^ This is an alarming issue and many authors have raised concerns about the under-diagnosis of physical illnesses in psychiatric patients.^14^,^15^

Some major physical illnesses detected in this study were hypertension (29.1%), respiratory diseases (15%), anaemia (12.5%), diabetes mellitus (10%) and liver diseases (5.8%), eye diseases (5%) and skin diseases (5%). Similar findings have been reported by Dixon *et* al.^16^ In another study, the odds of having diabetes, lung diseases and liver problems were found to be elevated.^17^ In India, some earlier studies done in the elderly population have shown similar findings.^18^,^19^

This study has demonstrated that a quick history checklist and physical examination can be helpful in detecting common physical illnesses of psychiatric patients. The fact that the number of patients with physical illnesses increased with systematic assessment provides support for such an approach. Psychiatrists must make sincere efforts to overcome their sense of inadequacy in this field. It would save time and money for both the patient and the healthcare system. A training programme for psychiatrists in common physical illnesses is warranted.

It was also observed in this study that a significantly higher percentage of subjects with freshly diagnosed physical illnesses were from the less educated and low-income socio-economic strata, although this finding of association may not have causative significance. It can be argued that in developing countries such as India, poverty breeds illness and finding a significantly higher degree of physical illness in patients from a low socioeconomic status is expected. Psychiatrists should remain vigilant so that common physical illnesses may not remain undetected in their clinical setting. Special attention should be paid while examining poor, less educated and unemployed persons. Improved detection and early treatment of physical illnesses in psychiatric patients will have a significant impact on their psychosocial functioning and quality of life. Patients often seek treatment for symptoms of disorders that are diagnosed as co-morbid, rather than principal conditions.

In the present study, the patients were taken from a psychiatric OPD service only. The findings of this study cannot be generalized to the entire clinic population as a significant number of patients were excluded from the study either due to unwillingness to participate or for dropping out. These findings need to be confirmed by community-based studies. Another limitation of our study is that smoking, alcohol and obesity have not been assessed, which are common in psychiatric populations and are independent risk factors for physical illnesses.
